# Functional Diversity in Ferns Is Driven by Species Richness Rather Than by Environmental Constraints

**DOI:** 10.3389/fpls.2020.615723

**Published:** 2021-01-11

**Authors:** Daniela Aros-Mualin, Sarah Noben, Dirk N. Karger, César I. Carvajal-Hernández, Laura Salazar, Adriana Hernández-Rojas, Jürgen Kluge, Michael A. Sundue, Marcus Lehnert, Dietmar Quandt, Michael Kessler

**Affiliations:** ^1^ Department of Systematic and Evolutionary Botany, University of Zurich, Zurich, Switzerland; ^2^ Swiss Federal Research Institute WSL, Birmensdorf, Switzerland; ^3^ Instituto de Investigaciones Biológicas, Universidad Veracruzana, Veracruz, México; ^4^ Centro de Investigación de la Biodiversidad y Cambio Climático (BioCamb) e Ingeniería en Biodiversidad y Recursos Genéticos, Facultad de Ciencias de Medio Ambiente, Universidad Tecnológica Indoamérica, Quito, Ecuador; ^5^ Department of Geography, Philipps University Marburg, Marburg, Germany; ^6^ The Pringle Herbarium, Department of Plant Biology, University of Vermont, Burlington, VT, United States; ^7^ Department of Geobotany and Botanical Garden, Herbarium, Martin-Luther-Universität Halle-Wittenberg, Halle, Germany; ^8^ Nees-Institute for Biodiversity of Plants, University of Bonn, Bonn, Germany

**Keywords:** functional diversity, community assembly, species richness, ferns, elevational gradient, environmental filtering, morphological diversity, niche packing

## Abstract

Functional traits determine how species interact with their abiotic and biotic environment. In turn, functional diversity describes how assemblages of species as a whole are adapted to their environment, which also determines how they might react to changing conditions. To fully understand functional diversity, it is fundamental to (a) disentangle the influences of environmental filtering and species richness from each other, (b) assess if the trait space saturates at high levels of species richness, and (c) understand how changes in species numbers affect the relative importance of the trait niche expansion and packing. In the present study, we determined functional diversity of fern assemblages by describing morphological traits related to resource acquisition along four tropical elevational transects with different environmental conditions and species richness. We used several functional diversity indices and their standardized effect size to consider different aspects of functional diversity. We contrasted these aspects of functional diversity with climate data and species richness using linear models and linear mixed models. Our results show that functional morphological trait diversity was primarily driven by species richness and only marginally by environmental conditions. Moreover, increasing species richness contributed progressively to packing of the morphological niche space, while at the same time decreasing morphological expansion until a saturation point was reached. Overall, our findings suggest that the density of co-occurring species is the fundamental driving force of morphological niche structure, and environmental conditions have only an indirect influence on fern resource acquisition strategies.

## Introduction

Studies of functional diversity have been increasingly used to understand community assembly processes (Petchey and Gaston, 2006; Mouchet et al., 2010; Lamanna et al., 2014; Tanaka and Sato, 2015; Pigot et al., 2016). Functional diversity measures what organisms do, rather than their taxonomic identity, and thus considers the functional complementarity and redundancy of co-occurring species (Diaz and Cabido, 2001; Petchey and Gaston, 2006). Functional diversity can be understood in terms of the Hutchinson’s niche concept (Hutchinson, 1957), i.e., by defining the distribution of species in a functional space whose axes are traits directly or indirectly linked to a function (Rosenfeld, 2002). This approach depends critically on the choice of traits, and thus, making an informed selection based on ecological function is vital.

Functional traits are morphological, anatomical, physiological, reproductive, or behavioral characteristics that affect how a species adapts to and interacts with its abiotic and biotic environment. Anatomical and physiological traits described by the leaf economic spectrum are often studied in plants (Bruelheide et al., 2018), although difficult to sample in large-scale studies. Among ferns, morphological traits such as life-form and plant size are widely used for describing functional diversity (Kluge and Kessler, 2007; Tanaka and Sato, 2015; Carvajal-Hernández et al., 2018; Nitta et al., 2020).

Also, because trait functionality has different aspects, it is necessary to use complementary indices to gain a better sense of which rules govern assembly processes (Mouchet et al., 2010). For instance, trait volume represents the amount of trait space covered by an assemblage. How evenly are species distributed in the trait space will relate directly to how effectively a community utilizes the entire range of resources available. Finally, density relates to how packed the species are inside this volume, indicating the degree of niche differentiation and resource competition.

A simple conceptual model of ecological assembly states that once a species arrives at a site, it first passes through an abiotic filter (i.e., environmental filtering) that determines which morphological and physiological traits allow a species to persist under a given set of ecological conditions (Weiher and Keddy, 1995). Extreme environments may select for a limited number of traits that allow persistence in those particular conditions. In these scenarios, co-occurring species will converge functionally (Grime, 2006), leading to more densely packed areas against others merely empty in the morphospace, and reducing how evenly traits are distributed. Moreover, increasing species packing is more likely to result in a smaller volume than expected by chance. Species next pass through a biotic filter of the competition of coexisting species, facilitation, or shared enemies. In particular, competition may lead to a divergence in functional strategy (limiting similarity theory; MacArthur and Levins, 1967), resulting in increasing trait volume with increasing species richness, leading to lower densities and species being more evenly distributed. Facilitation and shared enemies can result in highly variable effects on species traits in trait space, with patterns that can prove difficult to predict (Garzon-Lopez et al., 2015; Michalet and Pugnaire, 2016).

Ultimately, changes in trait volume reflect two complementary processes, functional niche expansion and niche packing (Swenson and Weiser, 2014). When species are drawn at random from a species pool, at low levels of species richness, trait volume is likely to increase as new species are added because they have trait combinations outside of the previously occupied niche space. At higher levels of species richness, additional species are more likely to fall within the already occupied trait space, increasing species packing (Pigot et al., 2016). The processes of functional niche expansion and packing have been widely studied for animals (Ricklefs and O’Rourke, 1975; Ricklefs and Cox, 1977). For example, in Andean birds, high species richness is mainly associated with functional niche packing, presenting a clear saturation pattern of functional diversity (Pigot et al., 2016). Patterns of functional niche expansion and packing are less well-understood in plants, but Swenson and Weiser (2014) found an increase of niche volume and packing with species richness in tree assemblages of eastern North America, without saturation of functional diversity.

Ferns and lycophytes (henceforth for simplicity called ferns) are a useful study group to assess biodiversity patterns because of their global distribution, contributing up to 70% of the species richness to local tropical floras, and because they have a reasonably high but manageable species richness (Kreft et al., 2010). In particular, being spore-dispersed reduces their dependence on biotic dispersal vectors, while enabling them to easily colonize suitable habitats, so that the confounding effects of dispersal limitation on assembly processes are reduced (Wolf et al., 2001; Karger et al., 2014). Previous studies on fern functional diversity along elevational gradients found that morphological trait diversity mirrors species richness patterns. In Costa Rica and Mexico, species richness and trait diversity show a hump-shaped pattern with maximum values at mid-elevation, Japan a monotonic decline, and the island of Moorea in French Polynesia a linear increase for epiphytes (Kluge and Kessler, 2007; Tanaka and Sato, 2015; Carvajal-Hernández et al., 2018; Nitta et al., 2020). Using a null model to exclude the influence of species richness, Kluge and Kessler (2007) found that for epiphytic ferns in Costa Rica, mid-elevations had higher than expected trait diversity, whereas upper and lower elevations maintained lower diversity. They interpreted this to reflect environmental filtering at the extremes of the gradient and biotic filtering in the middle. Similar conclusions were drawn for high elevations in Japan (Tanaka and Sato, 2015). However, these studies are limited to single transects, and because patterns of species richness and environmental variation (temperatures, precipitation, etc.) are closely correlated, it is challenging to truly isolate the effects of species richness and environment from each other. In contrast, if several transects are compared, different levels of species richness can be found at similar elevations and under similar ecological conditions, potentially allowing for disentangling their respective effects on community composition.

In the present study, we compared four tropical elevational transects with different environmental characteristics and species richness, including some of the most diverse fern assemblages worldwide in Ecuador and New Guinea, but also much poorer ones in Mexico and Uganda. We aimed to disentangle the influence of species richness and environment on functional diversity and asses the relative importance of functional expansion or packing with changes in species richness, by assessing morphological traits associated with resource acquisition. Specifically, we tested the following hypotheses:

H1: Functional diversity is mainly determined by (a) biotic filtering (measured as species richness), or (b): by environmental filtering.
H2: At low levels of species richness, increasing species numbers leads to an increase in trait volume, whereas at high levels of species richness, it leads to an increase of the density in the already occupied trait volume.

## Materials and Methods

### Transect Selection and Vegetation Sampling

**Figure 1:**
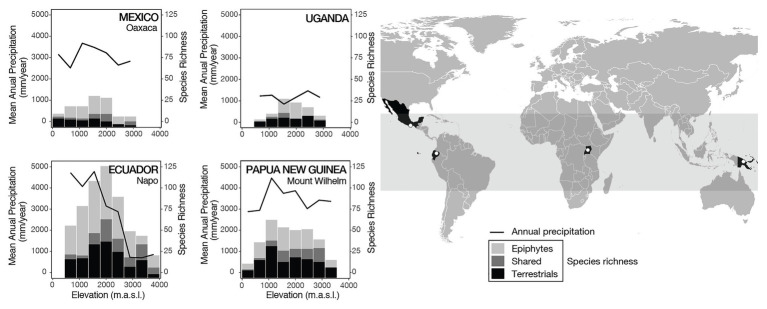
Annual precipitation (continuous lines) and fern species richness (bars) along the four tropical elevational gradients. The total number of species richness is divided into epiphytes (light gray), terrestrials (black), and species that were found as both epiphytes and terrestrials (dark gray). The gray band in the world map indicates the area between the tropics of Capricorn and Cancer.

Field surveys were conducted in 2009–2014 along elevational gradients in Ecuador (500–4,050 m), Mexico (300–3,000 m), Papua New Guinea (200–3,700 m), and Uganda (700–3,200 m), with all transects presenting a hump-shaped relationship with elevation (Figure 1). These transects together represent three major tropical biogeographic areas (America, Africa, and Asia-Oceania) with different levels of regional species richness (Weigand et al., 2020) and widely differing climates, allowing for the statistical separation of the effects of species richness from climate. In the field, we recorded epiphytic and terrestrial fern species in a total of 116 plots of 20 × 20 m^2^ each. Plots were placed in natural slope forests avoiding disturbed vegetation as well as ravines, ridges, rock faces, or other local microhabitats. Every fern species on each transect (but not in each plot) was collected and stored in a herbarium for later identification and to assess morphological traits. Epiphytes were identified and counted with binoculars, looking for fallen branches on the ground, climbing trees, and cutting selected branches. The abundance of each species was quantified, but we only used presence/absence data for our analyses. Detailed descriptions of field methods can be found in earlier publications (Kessler and Bach, 1999; Kluge and Kessler, 2007; Karger et al., 2014, 2015). Because of habitat availability and accessibility, not all elevational transects started at the same elevation, nor were the plots located at the same elevations. For the analyses, we defined intervals of 500 m, starting with an interval at 0–400 m (Table 1). Each interval included four plots per transect and all species recorded in those four plots were combined in a species list per interval, which formed the basis for the analyses. To assess if varying sampling completeness might influence our results, we used the Chao richness estimator (Chao, 1987) in EstimateS (Colwell, 2013) to estimate the total number of species in each interval. Estimated richness values were 1.6–90% (median 21%) higher than observed species counts (Supplementary Table S1), indicating that species sampling was not even between intervals. However, linear regression analyses of the observed vs. the estimated species numbers gave R^2^ values of 0.84–0.96 for the individual transects and of 0.93 overall (Supplementary Figure S1), showing that sampling incompleteness was roughly evenly distributed across our dataset and did not strongly influence our results based on the observed species numbers. We did not conduct our analyses with estimated species numbers because these are often biased (Herzog et al., 2002) and because our trait measurements were based on the actual species records and cannot be extrapolated.

**Table 1:** Numbers of species in each elevational interval and in total along the four study transects.

Interval	Elevation (m)	Number of species			
		Ecuador	Mexico	Papua New Guinea	Uganda
I	0–400	-	14	15	-
II	400–900	56	22	38	8
III	900–1,400	77	22	62	14
IV	1,400–1,900	104	33	54	31
V	1,900–2,400	120	31	51	26
VI	2,400–2,900	87	11	52	21
VII	2,900–3,400	50	11	47	12
VIII	3,400–3,900	45	-	19	-
IX	3,900–4,400	24	-	-	-
Total number of plots		32	28	32	24
Total species number		343	97	198	76

We established four plots in each elevational interval along each transect. There were in total 29 elevational intervals counting all transects together.

### Morphological Traits

We included morphological traits (hereafter “morphotraits”) related to resource utilization, i.e., light, water, and nutrient uptake, because these can be linked directly to resource competition between species. From the list of functional morphotraits of ferns of Kluge and Kessler (2007), we selected the shape and size of the rhizome and blades, laminar texture (which they referred to as thickness), and the presence of hydathodes (Table 2). We did not use traits related to reproduction (e.g., presence of indusia) or indument type and density. Although they are undoubtedly functionally relevant, they are not directly linked to resource acquisition, and their impact, if any, on competitive interactions is unclear. Consequently, if we included them, we would be mixing different adaptive and selective realms. Traits were compiled primarily from the relevant literature (e.g., Holttum, 1991; Stolze and Pacheco, 1994; Verdcourt et al., 1999/2008; Mickel and Smith, 2004), and missing information was garnered by the study of the herbarium specimens. Since laminar texture exhibits intraspecific variability, to avoid a pretense to accuracy, we used a broad character state definition as described in Table 2. Lamina length was calculated as the mean of the values reported in the literature or measured from the herbarium samples.

**Table 2:** Morphological traits of ferns used in this study.

Characteristic	Description	Class	Source	Scale of measurement
Rhizome type	Absent/unknown	0	a	Categorical
	Upright to short-creeping	1	a	
	Creeping	2	a	
	(stout) tree-like	3	a	
Laminar dissection	Entire	0	a	Ordinal
	Once-pinnate (to once-pinnate-pinnatifid)	1	a	
	Twice‐ or more-pinnate	2	a	
Laminar texture	Thin (membranaceous to thin herbaceous)	1	b	Ordinal
	Medium (herbaceous to paper-like)	2	b	
	Thick (thick paper-like to very coriaceous, stiff)	3	b	
Laminar length			b	Continuous
Hydathodes	Absent	0	a	Binary
	Present	1	a	

The information source is given as: (a) literature information or (b) literature information checked by own observations.

### Phylogenetic Signal

To evaluate the covariance of the evolutionary and morphological patterns, we tested if the selected morphotraits showed a phylogenetic signal. Since our focus was on functional diversity, phylogenetic signal was estimated based on all traits, rather than individual traits. Because the traits chosen are of mixed data types (categorical, continuous, and ordinal), we first computed a principal coordinate analysis (PCoA) based on a Gower’s dissimilarity matrix between all pairs of species and used the first and the second principal coordinates (which together explained 51% of the variance). We estimated Blomberg’s K using K statistics (R package “Picante”; Blomberg et al., 2003) that measures the phylogenetic signal of a trait by comparing the observed signal against a randomly evolving signal on the phylogeny. Trait conservatism is reflected by K values close to or larger than one, whereas trait convergence is reflected by values closer to zero. To assess the significance of the K statistics, we created a null model and randomly shuffled the taxa labels 999 times across the tips of the phylogeny, calculating K statistics for each randomization and comparing it to the variance of phylogenetically independent contrasts of the observed data.

### Functional Diversity

To quantify the morphological trait space (hereafter “morphospace”) occupied by the assemblages of each elevational interval, we used five functional diversity indices (Supplementary Figure S2) using the first five axes (80–84% of the variance) of the PCoA computed previously for the phylogenetic analyses. We calculated the morphovolume using two complementary indices: functional richness (FRic), which uses a minimum convex polygon for calculating the volume (R package “FD”; Villéger et al., 2008), and hypervolume (Hyp; R package “hypervolume”; Blonder et al., 2017) that after a multidimensional kernel density estimation procedure, delineates the boundaries of the *n*-dimensional volume. FRic is sensitive to outliers but could be computed with five PCoA axes and was also used for estimating packing and expansion along the transects as specified below, whereas Hyp is robust to outliers, but its calculation is complex so that it could only be computed with the first two PCoA axes. Next, we calculated functional evenness (FEve), which describes the evenness of distribution in the morphospace (R package “FD”; Villéger et al., 2008). Finally, we calculated morphodensity by the mean Euclidean distance between nearest neighbors (mNND) and its standard deviation (sdNND).

Since some of the indices used are known to be sensitive to species richness (FRic, NND, and SDNN; Mason et al., 2013), we calculated the standardized effect size (SES) for each metric with a null model analysis for which the species names in the morphological trait data were randomized (*n* = 5,000 replicates for all indices, except Hyp with *n* = 500). This approach is known as “independent swap” and was preferred to randomizing the species elevation distributions because it maintains the observed patterns of richness and range contiguity as well as trait co-variance and overall phenotypes (Swenson, 2014).

To understand the relative contribution of trait packing and expansion to species richness changes along the transects, we quantified niche expansion and niche packing between adjacent elevational bands. Because species richness presented a hump-shaped pattern, we defined a directionality from the elevation’s extremes to where the highest number of species was reached. Then, we calculated the percentage of additional species between the poorer elevation (E2) and a volume the size E2 inside the richer elevation (E1), together with the number of species outside of that volume. Because assemblages may differ in the shape or position of occupied volume, we developed an algorithm that, implementing a grid search, calculates the percentage of species in E1 that can be contained within a minimum convex polygon of a volume equivalent to that of E2. We then proceeded to remove species until the volume of E1 was equivalent to or in a vicinity of that occupied by E2. Thus, the number of species removed from E1 during the simulation represents the increase in richness due to trait expansion, whereas the number of species retained at the end of the simulation minus the ones in E2 represents the extra richness accommodated through trait packing (Pigot et al., 2016). Niche packing and expansion were calculated for the whole dataset and epiphytic species separately but not for terrestrial species due to low variation in species richness across the transects.

### Hypothesis Testing

We explored the relationship between the functional diversity indices and climate to test whether environmental conditions drove the functional morphology of these plants. We further used species richness as an indicator for competition for niche space, to assess the biotic component of filters of community composition. We analyzed epiphytic and terrestrial species separately because of their niche and functional differences (Kluge and Kessler, 2007). We used one microclimatic variable, namely, epiphytic bryophyte cover on branches of trees averaged by the elevational band as a proxy for air humidity (Karger et al., 2012), and four macroclimatic variables from CHELSA V1.2 (Karger et al., 2017, 2018), namely, mean annual temperature (MAT), temperature seasonality (TS), annual precipitation (AP), and precipitation seasonality (PS). Seasonality is a measure of change over the course of the year. The larger the percentage, the greater the variability, and requiring species to adapt to varying environmental conditions.

To assess the difference between handling all transects together or separately, linear mixed model analyses (LMM) were performed for all data, and linear models for each transect separately. Because the number of data points for each transect was small, we were unable to use all the climate data we used below, so elevation was used together with species richness against all indices.

To assess the first hypothesis, we contrasted climate data and species richness against all indices, as well as each SES with LMM using transects as a random factor. Additionally, we contrasted climate against species richness to consider an indirect effect over the indices. Implementing a structural equation modeling analysis to account for direct and indirect influences over functional diversity was impossible due to our limited number of sample sites. To test the second hypothesis, we implemented logistic regressions to contrast packing values (since they are expressed as percentages) and species richness using the transects as a random factor.

All models were calculated with the lmer4 package in R (Bates et al., 2015). For model selection, we used the dredge function in the R “MuMIn” package (Barton, 2018). Model fit was compared using the bias-corrected Akaike’s Information Criterion (AICc), and the relative importance of a variable across all models was obtained by summing its AICc weights in all competing models with ΔAICc < 2. The amount of variation explained by the fixed effects of each linear mixed model was calculated using Nakagawa and Schielzeth’s marginal R^2^ (Nakagawa and Schielzeth, 2013) from the MuMIn package in R (Barton, 2018). The package provides a marginal R^2^ that corresponds to the variance explained by the fixed effects and a conditional R^2^ that corresponds to the variance explained by the fixed and random effects together. All statistical analyses were implemented in the software R v. 3.4.0 (R Core Team, 2018).

## Results

The K statistics for a phylogenetic signal of all traits together revealed K values of 0.017 for PCoA axis 1 and 0.011 for axis 2 (both values with *p* < 0.001), indicating a convergent pattern of evolution with low phylogenetic signal.

The indices’ relation with species richness and elevation exhibited considerable variation for the individual transects, compared to the analysis done with all the transects together (Table 3), demonstrating that trends could be misinterpreted when studying single transects.

**Table 3:** Regression analyses of fern and lycophyte functional diversity in relation to species richness and elevation along four tropical elevational transects.

	Transect	Species richness	Elevation	R^2^
FRic	ECU	0.72	0.28	0.89^**^
	MX	0.65	0.35	0.78^*^
	PNG	0.73	0.27	0.90^**^
	UG	0.78	0.22	0.80^*^
	*Total*	0.71	0.29	0.85^**^
Hyp	ECU	0.76	0.24	-
	MX	0.50	0.50	0.72^*^
	PNG	0.75	0.25	0.58^*^
	UG	0.21	0.79	-
	*Total*	0.68	0.32	0.39^**^
FEve	ECU	0.50	0.50	0.95^**^
	MX	0.63	0.37	-
	PNG	0.79	0.21	0.64^*^
	UG	0.60	0.40	0.68^*^
	*Total*	1.00	0.00	0.68^**^
mNND	ECU	0.50	0.50	0.94^**^
	MX	0.69	0.31	0.78^**^
	PNG	0.72	0.27	0.76^**^
	UG	0.75	0.25	0.72^*^
	*Total*	1.00	0.00	0.89^**^
sdNND	ECU	0.70	0.30	0.56^*^
	MX	0.68	0.32	0.57^*^
	PNG	0.50	0.50	-
	UG	0.31	0.69	-
	*Total*	1.00	0.00	0.64^**^

The variable importance is calculated from the best models weighted on AIC values (ΔAIC < 2). Explained variances of the overall best-fitting model are provided and the fixed effects included are marked by a point (·). All calculations were made for each transect separately: Ecuador (ECU), Mexico (MX), Papua New Guinea (PNG), and Uganda (UG), and all together (Total), with the transects as a random factor. The functional diversity indices characterize the volume as functional volume (FRic) and hypervolume (Hyp), evenness (FEve), and density as the mean (mNND) and the standard deviation (sdNND) of the nearest neighbor distance. The fixed effect of the regression models was logarithmic for species richness and linear for elevation. FRic, mNND, and sdNND were log-transformed.

* *p* < 0.05.

** *p* < 0.005^*^.

**Figure 2:**
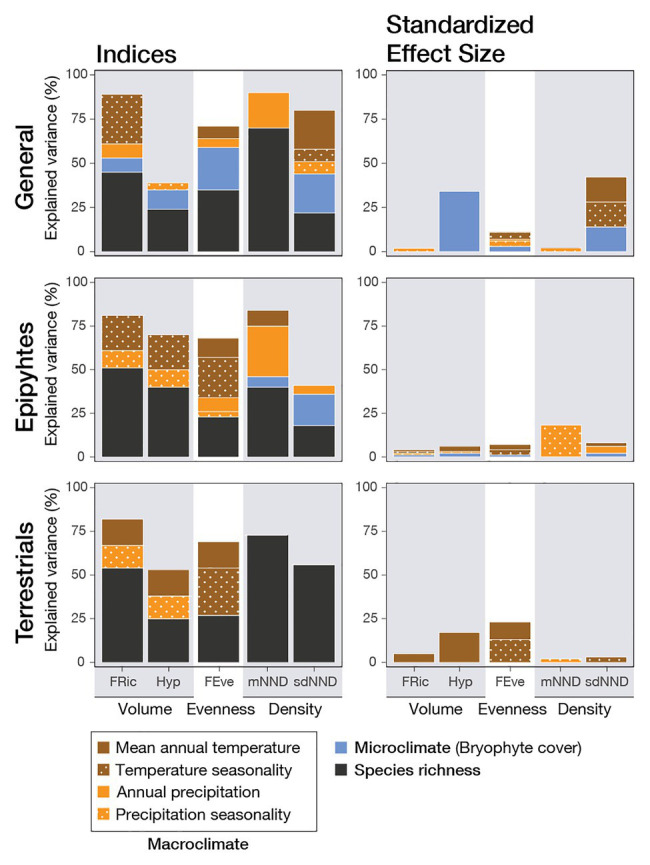
Variable importance of environmental factors and species richness for five functional diversity indices and their standardized effect sizes (SES). Values were obtained from the best linear mixed models (LMM) explaining fern and lycophyte functional diversity across four tropical elevational transects, weighted on AICc values (ΔAICc < 2). LMM used a set of different explanatory variables based on species richness, one microclimatic variable (bryophyte cover) and four macroclimatic variables (mean annual temperature, temperature seasonality, annual precipitation, and precipitation seasonality), using the transects as a random factor. The functional diversity indices characterize the volume as functional volume (FRic) and hypervolume (Hyp), evenness (FEve), and density as the mean (mNND) and the standard deviation (sdNND) of the nearest neighbor distance. The LMM were calculated for all data together (general) and then separated between epiphytic and terrestrial ferns. Explained variance was obtained as the mean R^2^ among all ΔAICc < 2 models.

Generally speaking, functional diversity given by all indices reached the strongest correlations with species richness; these became even more evident when separated between epiphytes and terrestrials (Figure 2). Once the influence of species richness was removed by calculating the standardized effect size, explained variances decreased, showing that, with some exceptions, the environment had a minor influence. When all species were analyzed together, the strongest correlations occurred with variables related to temperature and microclimate. The same was seen in terrestrials, whereas epiphytes were more strongly correlated with precipitation. Conversely, species richness by itself, when correlated to environmental variables, was most closely associated with water-related variables, and bryophyte cover was the most crucial variable for explaining the richness of epiphytes, terrestrials, and merged data (Table 4; Supplementary Table S2).

**Table 4:** Variable importance of environmental factors influencing tropical fern and lycophyte species richness.

	BC	MAT	TS	AP	PS	R^2^	*χ* ^2^(3)
General	0.33	0.33	0	0	0.33	0.61	24.34^**^
Epiphytes	0.36	0.19	0.08	0.24	0.13	0.51	21.86^**^
Terrestrials	0.38	0	0	0.25	0.37	0.54	13.57^**^

Values were obtained from the best linear mixed models (LMM) explaining species richness across four tropical elevational transects weighted by AICc values (ΔAICc < 2). LMM used a set of different explanatory variables based on one microclimatic variable (bryophyte cover BC) and four macroclimatic variables [mean annual temperature (MAT), temperature seasonality (TS), annual precipitation (AP), and precipitation seasonality (PS)]. LMM were calculated for all data together (general) and then separately for epiphytic and terrestrial ferns, using the transects as random factor. Zero values are due to the absence of the respective fixed effect in the best models (ΔAICc < 2). The R^2^ and *χ* ^2^ values (with 3 degrees of freedom) of the overall best-fitting model (determine by AICc) are provided and the fixed effects included are marked by a point (·).

** *p* < 0.005.

**Figure 3:**
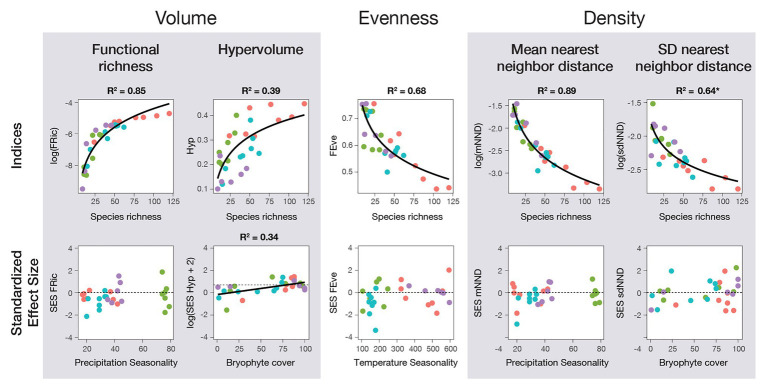
Fern and lycophyte functional diversity indices in relation to species richness and environmental variables for four tropical transects in Ecuador (red), Mexico (green), Papua New Guinea (blue), and Uganda (purple). Linear mixed models (LMM) were used to contrast climate data and species richness against all indices, as well as the standardized effect size (SES) of each using the transects as a random factor. Climatic data used was one microclimatic variable (bryophyte cover in tree branches; bryophyte cover) and four macroclimatic variables (mean annual temperature, temperature seasonality, annual precipitation, and precipitation seasonality). All R^2^ values are of LMM containing only species richness or the climatic data specified in the graph and were part of the selected models for the variable importance analysis (best fitting models, ΔAICc < 2). In contrast, values marked with (^*^) are LMM that were not selected but still have significantly explained variance.

Morphovolume, quantified using FRic and Hyp, showed a positive logarithmic relationship with species richness for all species, epiphytes, and terrestrials (Figures 2, 3). To a lesser degree, it was also correlated to environmental variables, although the direct correlation with species richness was stronger. There was a faint negative logarithmic relation between annual precipitation and FRic, and a positive linear correlation between bryophyte cover and Hyp, including all species. When separated by life-form, precipitation seasonality was most relevant for epiphytes, whereas there was a negative relation with annual mean temperature for terrestrials. Meanwhile, using the SES values, only Hyp still presented a correlation with the environment, specifically with bryophyte cover (Figure 3).

Functional evenness (FEve) was significantly negatively related to species richness in a logarithmic fashion for all species and when separated by life-form (Figure 3; Supplementary Figure S3). This suggests the formation of clusters of species with an increase in species richness. Even though species richness was more important than the other variables for all species together, temperature seasonality became as important when separated by epiphytes and terrestrials, and was still important in terrestrials when the SES of FEve was estimated (Figure 2).

Morphodensity, given by the mean (mNND) and standard deviation (sdNND) of the nearest neighbor distance, was best explained by species richness (Figure 2). The negative logarithmic correlations of both indices with species richness (Figure 3) translated into an increase in density. To a lesser degree, environmental factors also had some influence, which changed according to the index and data considered. For mNND, annual precipitation was the most important predictor for all species together and epiphytes, whereas terrestrials showed no correlation to environmental factors at all (Figure 2). SES of mNND showed a shallow relation to precipitation seasonality for epiphytes (Figure 2; Supplementary Figure S3). By contrast, sdNND for all species together and epiphytes showed a positive correlation with bryophyte cover, whereas terrestrials showed no relation to the environment (Figure 2; Supplementary Tables S3, S4). Even though SES of sdNND of all species together correlated with the environment, there was no correlation for any individual environmental variables. Moreover, when separated by life-form, there was no correlation with the environment at all (Figure 3).

**Figure 4:**
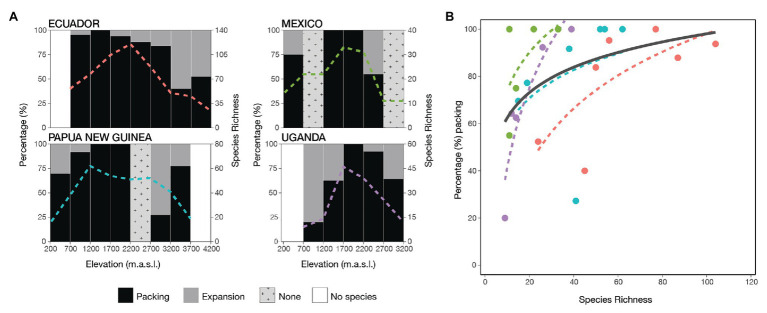
Morphoniche packing and expansion of ferns along four elevational gradients. **(A)** The percentage contribution of packing (black) and expansion (gray) between adjacent elevational bands, showing species richness by the colored dotted lines. If there is no variation in species richness between elevational steps, then it was not possible to calculate the indices which appears in light gray as none. **(B)** The relationship of packing percentage with the increase of species richness for all transects [ΔAICc = 28.9 *χ* ^2^(1) = 31.6, *p* < 0.001]. The transects are indicated by colors: Ecuador (red), Mexico (green), Papua New Guinea (blue), and Uganda (purple). The general trend is given in black and the transects are shown by the same color as in **(A)**.

Packing was positively logarithmically correlated with species richness (Figure 4B: ΔAICc = 28.9 *χ* ^2^(1) = 31.6, *p* < 0.001; Supplementary Figure S4: ΔAICc = 3.49 *χ* ^2^(1) = 11.4, *p* < 0.001), showing that there was an increase in volume at low levels of species richness. However, it was almost saturated at higher levels as a result of new species mostly being packed inside the pre-existing volume and thus increasing density.

**Figure 5:**
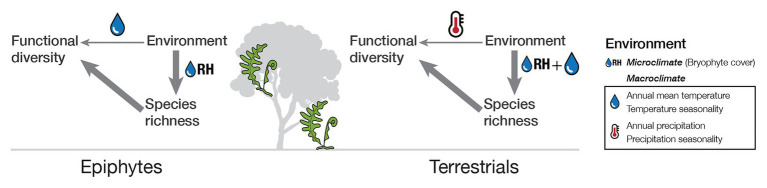
Schematic representation of the relationship of functional diversity to environmental conditions and species richness for tropical epiphytic and terrestrial ferns.

## Discussion

The first aim of our study was to separate the effects of species richness and environmental conditions in determining functional diversity in fern assemblages. Previous studies have not been able to make this distinction because they focused on single transects where species richness and environmental conditions are closely correlated, making it impossible to separate their influence on functional diversity (Kluge and Kessler, 2007; Tanaka and Sato, 2015; Carvajal-Hernández et al., 2018). In our study, when analyzing transects separately, we indeed found the same situation (Table 3). However, when all transects were analyzed in combination (Table 3), the models that best explain functional diversity were the ones that predominantly considered the effect of species richness (Figure 2). Further, when the effect of species richness was excluded by using the standardized effect size of each index (SES), functional diversity was only weakly correlated to environmental factors. It is also important to note that the traits used in this study showed low phylogenetic signal, so that the relationship between functional diversity and species richness is not directly determined by phylogenetic patterns but by convergent evolutionary patterns.

Regarding the second aim of our study, we found that the functional morphovolume nearly reached saturation with increasing species richness. Full saturation will be reached when all extreme possible combinations are realized, such that new species will increase redundancy or further partition niche space that is already occupied (Mayfield et al., 2010; Cadotte et al., 2011). We found that at low levels of species richness, in our case at the extremes of each transect, additional species mainly tended to increase the volume. In contrast, with increasing species richness toward the middle elevations, the addition of species was mainly *via* increased packing (Figure 4). Swenson and Weiser (2014) present similar results for tree assemblages in eastern North America, where both the volume and the degree of packing increased with increasing species richness. In their study of Andean birds, Pigot et al. (2016) also observed that high species richness is more strongly associated with a denser occupation of the trait morphospace than with increasing niche volume.

Looking more closely at the indices of niche packing, we found that the mean and standard deviations of the nearest neighbor distances as well as evenness decreased with increasing species richness. This points toward species being packed in some sections of the volume, whereas others are only sparsely occupied, which could be a result of co-variances between the studied traits so that not all trait combinations are possible. Moreover, an increase in evenness with species richness has often been interpreted as limits to phenotypic similarity (Kraft et al., 2008). Since evenness showed a decrease, our results provide no evidence for this limitation, as previously found for Andean birds (Pigot et al., 2016).

While we found that species richness was the strongest determinant of morphological diversity in our study assemblages, we also found that environmental factors played a minor role, especially when we distinguished between life-forms (Figure 2; SES of indices). For epiphytes, the exposure to frequent and even extended periods of drought is more common than in terrestrials living in the same habitat (Watkins et al., 2007). The higher up in the canopy, the stronger the light intensity and changes in air humidity, with little soil to act as a buffer. Even small changes in environmental conditions could have a considerable impact on plants living in the canopy (Petter et al., 2016). Our results are in accordance with this and reveal that trait diversity in epiphytes is mainly influenced by seasonality, in particular precipitation seasonality. For terrestrial ferns, temperature variables had the most substantial influence. Since there is high humidity in the tropics and soil provides a buffer for microclimatic changes, the most substantial variation endured by terrestrials would be given by changes in temperature. However, the pattern changes if, instead of examining the effect over functional trait diversity, the influence of environment over species richness is taken into account. Our results showed that although species richness showed similar patterns to functional diversity, the most crucial factor regardless of life-form was a microclimatic variable, namely, epiphytic bryophyte cover which we used as a proxy for air humidity. In fact, fern diversity is primarily driven by the energy-water balance, with water availability being the most limiting factor (Kreft et al., 2010; Qian et al., 2012; Khine et al., 2019; Weigand et al., 2020). Together this unveils an indirect effect of the environment through species richness over trait diversity (Figure 5).

Like any study of this kind, ours features methodological limitations. First, we were restricted to morphological traits that can readily be gathered for a large number of species (800 in total in our study) rather than physiological and anatomical traits such as leaf mass per area (LMA) as is typical in studies of plant functional traits (e.g., Bruelheide et al., 2018). There is no doubt that traits such as plant size or the spatial distribution of the rhizome system are adaptive and have a function in resource acquisition and interspecific competition. Still, our study might perhaps have obtained different results if we were to include a different set of traits. Yet, we consider this unlikely since a study of Brazilian ferns using traits of the leaf economic spectrum also found a strong signal of trait convergence along environmental gradients that is in accordance with our results (Costa et al., unpublished data). Second, the majority of our traits were scored as categorical or ordinal, partly because some traits were binary by nature, and partly because some have high infraspecific variability that is best captured in categories. This type of data can lead to loss of resolution, which might limit our inferences. However, we doubt that our main result of the predominance of species richness would be affected by a finer estimation of morphological diversity. More generally, ignoring intraspecific trait variability restricts our ability to differentiate if changes in the degree of packing were due to varying trait niche widths (i.e., finer specialization) or varying trait niche overlap (MacArthur, 1965). Intraspecific trait variability is relevant for plant communities at local scales and along short environmental gradients (Carmona et al., 2015; Siefert et al., 2015) and finer specialization may also underlie elevational diversity gradients (Hulshof et al., 2013). Indeed, previous studies of realized environmental niches among ferns have shown that these decrease with increasing species richness (Karger et al., 2015). Finally, our selected study areas are restricted to tropical environments. Yet, based on our understanding of global patterns of fern diversity (Weigand et al., 2020) and the fact that transect plot data can be used to extrapolate richness patterns across large geographical regions (Khine et al., 2019), we are confident that our transects are representative and that our results broadly apply to tropical continental fern assemblages. Whether they also apply to depauperate temperate and oceanic insular fern assemblages remains to be studied.

Taken together and bearing the above constraints in mind, our results reveal that high species richness contributes progressively to packing of the morphological niche space while at the same time increasing functional diversity. Moreover, at a given level of species richness, the environment did not strongly modify the morphological niche structure, even though in our case this was often under varied climatic conditions among transects belonging to different biogeographic regions with vastly distinct regional fern diversities (Kreft et al., 2010; Weigand et al., 2020). Since species richness itself is strongly influenced by environmental conditions (Wright, 1983; Hawkins et al., 2003; Kessler et al., 2011), there is an indirect influence of the environment on trait diversity. Still, the fundamental driving force of resource acquisition strategies in ferns appears to be the number of species.

## Data Availability Statement

The raw data supporting the conclusions of this article will be made available by the authors, without undue reservation.

## Author Contributions

MK, ML, and DQ conceived the ideas and designed the methodology. AH-R, CC-H, DA-M, DK, DQ, JK, MK, ML, MS, LS, and SN collected the data. DA-M, DK, and SN analyzed the data. DA-M and MK led the writing of the manuscript. All authors contributed to the article and approved the submitted version.

### Conflict of Interest

The authors declare that the research was conducted in the absence of any commercial or financial relationships that could be construed as a potential conflict of interest.
