# Supplementary material for: Functional Diversity in Ferns Is Driven by Species Richness Rather Than by Environmental Constraints
Source: Front Plant Sci. 2021 Jan 11;11:615723. doi: 10.3389/fpls.2020.615723 (PMC7829179; doi:10.3389/fpls.2020.615723)
Supplement: Supplementary file 1 [file Data_Sheet_1.docx]

**Supplementary material**

Table S1. Observed versus estimated species richness (%) of ferns and lycophytes along four elevational transects in the tropics, computed with Chao2 estimator. The transects are in Ecuador (ECU), Mexico (MX), Papua New Guinea (PNG), and Uganda (UG). Median: 20.5%, maximum value 90% and minimum 1.6%. The values were calculated as the difference between the estimated and observed value, divided by the observed value and multiply by 100. R^2^ and p values correspond to the linear regressions of observed versus estimated species richness.

| Elevation | ECU | MX | PNG | UG |
| --- | --- | --- | --- | --- |
| I | - | 8.9 | **90.0** | - |
| II | 62.6 | 44.3 | 53.5 | **1.6** |
| III | 10.2 | 4.9 | 22.4 | 32.1 |
| IV | 72.5 | 16.1 | 8.9 | 3.5 |
| V | 42.2 | 21.8 | 36.8 | 3.2 |
| VI | 17.8 | 20.5 | 19.8 | 12.5 |
| VII | 17.6 | 3.5 | 24.0 | 3.2 |
| VIII | 67.7 | - | 35.5 | - |
| IX | 48.8 | - | - | - |
| R^2^ | 0.84 | 0.93 | 0.89 | 0.96 |
| p | 0.0008 | 0.0003 | 0.0003 | 0.0004 |

Table S2. Species richness of ferns and lycophytes in relation to environmental variables for four elevational transects in the tropics. Linear mixed models were used to contrast climate data against species richness, using the transects as a random factor. Only the best models are listed, weighted on AIC values (ΔAIC<2, indicated by delta). Climatic data used was one microclimatic variable (bryophyte cover: Bryophyte) and four macroclimatic variables (mean annual temperature MAT, temperature seasonality TS, annual precipitation AP, and precipitation seasonality PS). All R^2^ values were calculated using Nakagawa and Schielzeth’s method (marginal (R^2^m) and conditional (R^2^c); Nakagawa & Schielzeth, 2013).

| Model |  | |  | df | | | AICc | | delta | | weight | | R^2^m | | R^2^c |  |
| --- | --- | --- | --- | --- | --- | --- | --- | --- | --- | --- | --- | --- | --- | --- | --- | --- |
| sp_rich (Merged data) | | ~ Bryophyte+log(MAT)+log(PS) | | | 6 | 257.69 | | 0.000 | | 0.341 | | 0.61 | | 0.75 | | |
|  |  | ~ Bryophyte+log(MAT)+log(AP)+log(PS) | | | 7 | 259.72 | | 2.027 | | 0.124 | | 0.62 | | 0.72 | | |
| sp_rich (Epiphytes) | | ~ Bryophyte+log(MAT)+log(PS) | | | 6 | 228.70 | | 0.000 | | 0.222 | | 0.51 | | 0.80 | | |
|  |  | ~ Bryophyte+log(AP) | | | 5 | 229.49 | | 0.794 | | 0.149 | | 0.30 | | 0.75 | | |
|  |  | ~ Bryophyte+log(TS)+log(AP) | | | 6 | 229.64 | | 0.941 | | 0.139 | | 0.40 | | 0.72 | | |
|  |  | ~ Bryophyte+log(MAT)+log(AP) | | | 6 | 229.91 | | 1.219 | | 0.121 | | 0.28 | | 0.77 | | |
| sp_rich (Terrestrials) | | ~ Bryophyte+log(AP)+log(PS) | | | 6 | 223.07 | | 0.000 | | 0.198 | | 0.54 | | 0.54 | | |
|  |  | ~ Bryophyte+log(PS) | | | 5 | 223.78 | | 0.718 | | 0.138 | | 0.44 | | 0.51 | | |
|  |  | ~ Bryophyte+log(AP) | | | 5 | 224.63 | | 1.565 | | 0.090 | | 0.22 | | 0.54 | | |
|  |  | ~ log(PS) | | | 4 | 224.70 | | 1.635 | | 0.087 | | 0.34 | | 0.41 | | |

Table S3. Functional diversity indices of ferns and lycophytes in relation to species richness and environmental variables for four elevational transects in the tropics. Linear mixed models were used to contrast climate data and species richness against all indices, as well as each standardized effect size (SES) using the transects as a random factor. Only the best models are listed, weighted on AIC values (ΔAIC<2, indicated by delta). The functional diversity indices characterize the volume as functional volume (FRic) and hypervolume (Hyp), evenness (FEve), and density as the mean (mNND) and the standard deviation (sdNND) of the nearest neighbor distance. Climatic data used was one microclimatic variable (bryophyte cover: Bryophyte) and four macroclimatic variables (mean annual temperature MAT, temperature seasonality TS, annual precipitation AP, and precipitation seasonality PS). All R^2^ values were calculated using Nakagawa and Schielzeth’s method (marginal (R^2^m) and conditional (R^2^c); Nakagawa & Schielzeth, 2013).

| Model | |  |  | df | | | AICc | | delta | | weight | | R^2^m | | R^2^c |  |
| --- | --- | --- | --- | --- | --- | --- | --- | --- | --- | --- | --- | --- | --- | --- | --- | --- |
| log(FRic) | ~ log(sp_rich) | | | | 4 | 42.94 | | 0.000 | | 0.231 | | 0.89 | | 0.89 | | |
|  | ~ log(PS)+log(sp_rich) | | | | 5 | 43.09 | | 0.155 | | 0.213 | | 0.9 | | 0.9 | | |
|  | ~ log(MAT)+log(sp_rich) | | | | 5 | 43.36 | | 0.423 | | 0.187 | | 0.9 | | 0.9 | | |
|  | ~ log(MAT)+log(PS)+log(sp_rich) | | | | 6 | 43.41 | | 0.471 | | 0.182 | | 0.91 | | 0.91 | | |
|  | ~ Bryophyte+log(MAT)+log(PS)+log(sp_rich) | | | | 7 | 44.59 | | 1.650 | | 0.101 | | 0.92 | | 0.92 | | |
|  | ~ log(TS)+log(sp_rich) | | | | 5 | 44.9 | | 1.961 | | 0.086 | | 0.89 | | 0.89 | | |
| Hyp | ~ log(sp_rich) | | | | 4 | -66.69 | | 0.000 | | 0.309 | | 0.29 | | 0.61 | | |
|  | ~ Bryophyte+log(sp_rich) | | | | 5 | -66.65 | | 0.043 | | 0.303 | | 0.3 | | 0.66 | | |
|  | ~ log(MAT)+log(sp_rich) | | | | 5 | -65.29 | | 1.400 | | 0.154 | | 0.38 | | 0.63 | | |
|  | ~ log(PS)+log(sp_rich) | | | | 5 | -64.79 | | 1.894 | | 0.120 | | 0.24 | | 0.61 | | |
|  | ~ log(TS)+log(sp_rich) | | | | 5 | -64.70 | | 1.988 | | 0.114 | | 0.33 | | 0.62 | | |
| FEve | ~ Bryophyter+log(sp_rich) | | | | 5 | -73.72 | | 0.000 | | 0.542 | | 0.53 | | 0.8 | | |
|  | ~ Bryophyte+log(PS)+log(sp_rich) | | | | 6 | -72.05 | | 1.676 | | 0.234 | | 0.57 | | 0.86 | | |
|  | ~ Bryophyte+log(TS)+log(sp_rich) | | | | 6 | -71.95 | | 1.773 | | 0.223 | | 0.6 | | 0.81 | | |
| log(mNND) | ~ log(sp_rich) | | | | 4 | -16.13 | | 0.000 | | 0.559 | | 0.87 | | 0.89 | | |
|  | ~ log(AP)+log(sp_rich) | | | | 5 | -14.38 | | 1.756 | | 0.232 | | 0.87 | | 0.89 | | |
|  | ~ log(TS)+log(sp_rich) | | | | 5 | -14.17 | | 1.966 | | 0.209 | | 0.87 | | 0.89 | | |
| log(sdNND) | ~ Bryophyte+log(MAT)+log(sp_rich) | | | | 6 | -9.62 | | 0.000 | | 0.391 | | 0.63 | | 0.63 | | |
|  | ~ Bryophyte+log(sp_rich) | | | | 5 | -9.41 | | 0.207 | | 0.353 | | 0.37 | | 0.56 | | |
|  | ~ log(sp_rich) | | | | 4 | -8.77 | | 0.854 | | 0.255 | | 0.27 | | 0.51 | | |
| SES FRic | ~ none | | | | 3 | 79.82 | | 0.000 | | 0.682 | | 0.00 | | 0.00 | | |
|  | ~ log(PS) | | | | 4 | 81.35 | | 1.527 | | 0.318 | | 0.04 | | 0.04 | | |
| SES log(Hyp+2) | ~ Bryophyte | | | | 4 | 54.46 | | 0.000 | | 0.431 | | 0.34 | | 0.34 | | |
|  | ~ none | | | | 3 | 94.20 | | 0.000 | | 0.186 | | 0.00 | | 0.03 | | |
| SES FEve | ~ log(PS)+log(TS) | | | | 5 | 94.43 | | 0.237 | | 0.165 | | 0.18 | | 0.18 | | |
|  | ~ Bryophyte+log(PS)+log(TS) | | | | 6 | 94.58 | | 0.377 | | 0.154 | | 0.26 | | 0.26 | | |
|  | ~ Bryophyte | | | | 4 | 95.02 | | 0.816 | | 0.124 | | 0.07 | | 0.19 | | |
|  | ~ log(TS) | | | | 4 | 95.08 | | 0.884 | | 0.120 | | 0.07 | | 0.07 | | |
|  | ~ log(PS) | | | | 4 | 95.73 | | 1.529 | | 0.087 | | 0.04 | | 0.04 | | |
|  | ~ log(AP) | | | | 4 | 95.76 | | 1.562 | | 0.085 | | 0.04 | | 0.04 | | |
|  | ~ Bryophyte+log(TS) | | | | 5 | 95.95 | | 1.755 | | 0.078 | | 0.13 | | 0.13 | | |
| SES mNND | ~ none | | | | 3 | 78.79 | | 0.000 | | 0.684 | | 0.00 | | 0.00 | | |
|  | ~ log(PS) | | | | 4 | 80.34 | | 1.552 | | 0.315 | | 0.04 | | 0.04 | | |
| SES sdNND | ~ Bryophyte+log(MAT)+log(TS) | | | | 6 | 84.74 | | 0.000 | | 1.000 | | 0.42 | | 0.42 | | |

Table S4. Functional diversity indices of epiphytic ferns and lycophytes in relation to species richness and environmental variables for four elevational transects in the tropics. Linear mixed models were used to contrast climate data and species richness against all indices, as well as each standardized effect size (SES) using the transects as a random factor. Only the best models are listed, weighted on AIC values (ΔAIC<2, indicated by delta). The functional diversity indices characterize the volume as functional volume (FRic) and hypervolume (Hyp), evenness (FEve), and density as the mean (mNND) and the standard deviation (sdNND) of the nearest neighbor distance. Climatic data used was one microclimatic variable (bryophyte cover: Bryophyte) and four macroclimatic variables (mean annual temperature MAT, temperature seasonality TS, annual precipitation AP, and precipitation seasonality PS). All R^2^ values were calculated using Nakagawa and Schielzeth’s method (marginal (R^2^m) and conditional (R^2^c); Nakagawa & Schielzeth, 2013).

| Models | |  | df | | | AICc | | delta | | weight | | R^2^m | | R^2^c | |
| --- | --- | --- | --- | --- | --- | --- | --- | --- | --- | --- | --- | --- | --- | --- | --- |
| log(FRic) | ~ log(sp_rich)+Bryophyte+log(PS) | | | 6 | 95.76 | | 0.000 | | 0.386 | | 0.83 | | 0.83 | |  |
|  | ~ log(sp_rich)+log(PS) | | | 5 | 96.83 | | 1.070 | | 0.226 | | 0.79 | | 0.80 | |  |
|  | ~ log(sp_rich)+log(TS) | | | 5 | 96.97 | | 1.204 | | 0.211 | | 0.80 | | 0.80 | |  |
|  | ~ log(sp_rich) | | | 4 | 97.33 | | 1.560 | | 0.177 | | 0.76 | | 0.79 | |  |
| Hyp | ~ log(sp_rich)+log(PS) | | | 5 | -68.89 | | 0.000 | | 0.430 | | 0.79 | | 0.79 | |  |
|  | ~ log(sp_rich) | | | 4 | -68.18 | | 0.706 | | 0.302 | | 0.75 | | 0.79 | |  |
|  | ~ log(sp_rich)+log(TS)+log(PS) | | | 6 | -67.94 | | 0.945 | | 0.268 | | 0.81 | | 0.81 | |  |
| FEve | ~ log(sp_rich)+log(TS) | | | 5 | -72.19 | | 0.000 | | 0.358 | | 0.66 | | 0.66 | |  |
|  | ~ log(sp_rich)+log(MAT)+log(TS)+log(AP) | | | 7 | -72.00 | | 0.187 | | 0.326 | | 0.73 | | 0.73 | |  |
|  | ~ log(sp_rich)+log(MAT)+log(TS) | | | 6 | -70.65 | | 1.535 | | 0.166 | | 0.68 | | 0.68 | |  |
|  | ~ log(sp_rich)+log(TS)+log(PS) | | | 6 | -70.45 | | 1.733 | | 0.150 | | 0.67 | | 0.67 | |  |
| log(mNND) | ~ log(sp_rich)+log(AP) | | | 5 | 11.41 | | 0.000 | | 0.363 | | 0.83 | | 0.83 | |  |
|  | ~ log(sp_rich) | | | 4 | 11.93 | | 0.525 | | 0.279 | | 0.81 | | 0.81 | |  |
|  | ~ log(sp_rich)+log(MAT)+log(AP) | | | 6 | 12.41 | | 1.006 | | 0.219 | | 0.85 | | 0.85 | |  |
|  | ~ log(sp_rich)+Bryophyte+log(AP) | | | 6 | 13.33 | | 1.926 | | 0.139 | | 0.84 | | 0.84 | |  |
| sdNND | ~ log(sp_rich)+Bryophyte | | | 5 | -114.55 | | 0.000 | | 0.714 | | 0.47 | | 0.47 | |  |
|  | ~ log(sp_rich)+Bryophyte+log(AP) | | | 6 | -112.72 | | 1.828 | | 0.286 | | 0.49 | | 0.49 | |  |
| SES FRic | ~ none | | | 3 | 99.83 | | 0.000 | | 0.370 | | 0.00 | | 0.37 | |  |
|  | ~ Bryophyte | | | 4 | 101.42 | | 1.594 | | 0.167 | | 0.03 | | 0.37 | |  |
|  | ~ log(PS) | | | 4 | 101.52 | | 1.696 | | 0.158 | | 0.07 | | 0.40 | |  |
|  | ~ log(TS) | | | 4 | 101.56 | | 1.729 | | 0.156 | | 0.09 | | 0.30 | |  |
|  | ~ log(MAT) | | | 4 | 101.67 | | 1.838 | | 0.147 | | 0.02 | | 0.42 | |  |
| SES_Hyp | ~ none | | | 5 | 79.72 | | 0.000 | | 0.307 | | 0.00 | | 0.00 | |  |
|  | ~ Bryophyte + log(MAT) | | | 6 | 80.52 | | 0.807 | | 0.205 | | 0.17 | | 0.17 | |  |
|  | ~ log(MAT) | | | 3 | 80.64 | | 0.931 | | 0.192 | | 0.06 | | 0.06 | |  |
|  | ~ log(PS) | | |  | 80.95 | | 1.237 | | 0.165 | | 0.05 | | 0.05 | |  |
|  | ~ Bryophyte | | | 6 | 81.44 | | 1.726 | | 0.130 | | 0.03 | | 0.03 | |  |
| SES FEve | ~ none | | | 3 | 91.79 | | 0.000 | | 0.333 | | 0.00 | | 0.48 | |  |
|  | ~ log(MAT)+log(TS) | | | 5 | 93.03 | | 1.233 | | 0.180 | | 0.25 | | 0.57 | |  |
|  | ~ log(TS) | | | 4 | 93.16 | | 1.367 | | 0.168 | | 0.11 | | 0.44 | |  |
|  | ~ log(MAT) | | | 4 | 93.18 | | 1.382 | | 0.167 | | 0.03 | | 0.52 | |  |
|  | ~ Bryophyte | | | 4 | 93.38 | | 1.582 | | 0.151 | | 0.03 | | 0.48 | |  |
| SES mNND | ~ log(PS) | | | 4 | 85.86 | | 0.000 | | 1.000 | | 0.18 | | 0.18 | |  |
| SES sdNND | ~ none | | | 3 | 76.06 | | 0.000 | | 0.325 | | 0.00 | | 0.00 | |  |
|  | ~ log(AP) | | | 4 | 76.22 | | 0.165 | | 0.299 | | 0.09 | | 0.09 | |  |
|  | ~ log(MAT) | | | 4 | 76.98 | | 0.924 | | 0.205 | | 0.06 | | 0.06 | |  |
|  | ~ Bryophyte + log(AP) | | | 5 | 77.33 | | 1.273 | | 0.172 | | 0.15 | | 0.15 | |  |

Table S5. Functional diversity indices of terrestrial ferns and lycophytes in relation to species richness and environmental variables for four elevational transects in the tropics. Linear mixed models were used to contrast climate data and species richness against all indices, as well as each standardized effect size (SES) using the transects as a random factor. Only the best models are listed, weighted on AIC values (ΔAIC<2, indicated by delta). The functional diversity indices characterize the volume as functional volume (FRic) and hypervolume (Hyp), evenness (FEve), and density as the mean (mNND) and the standard deviation (sdNND) of the nearest neighbor distance. Climatic data used was one microclimatic variable (bryophyte cover: Bryophyte) and four macroclimatic variables (mean annual temperature MAT, temperature seasonality TS, annual precipitation AP, and precipitation seasonality PS). All R^2^ values were calculated using Nakagawa and Schielzeth’s method (marginal (R^2^m) and conditional (R^2^c); Nakagawa & Schielzeth, 2013).

| Models |  | df | AICc | | delta | | weight | | R^2^m | | R^2^c | |
| --- | --- | --- | --- | --- | --- | --- | --- | --- | --- | --- | --- | --- |
| log(FRic) | ~ log(sp_rich) | 4 | | 79.88 | | 0.000 | | 0.478 | | 0.81 | | 0.81 |
|  | ~ log(sp_rich)+log(MAT) | 5 | | 80.95 | | 1.063 | | 0.281 | | 0.82 | | 0.82 |
|  | ~ log(sp_rich)+log(PS) | 5 | | 81.24 | | 1.361 | | 0.242 | | 0.82 | | 0.82 |
| Hyp | ~ log(sp_rich)+log(MAT)+log(PS) | 6 | | -63.48 | | 0.000 | | 0.514 | | 0.66 | | 0.66 |
|  | ~ log(sp_rich)+log(MAT) | 5 | | -63.37 | | 0.116 | | 0.486 | | 0.62 | | 0.64 |
| FEve | ~ log(sp_rich)+log(MAT)+log(TS) | 6 | | -77.29 | | 0.000 | | 0.542 | | 0.71 | | 0.71 |
|  | ~ log(sp_rich)+log(TS) | 5 | | -76.95 | | 0.340 | | 0.458 | | 0.67 | | 0.67 |
| log(mNND) | ~ log(sp_rich) | 4 | | 10.90 | | 0.000 | | 1.000 | | 0.73 | | 0.80 |
| sdNND | ~ log(sp_rich) | 4 | | -131.84 | | 0.000 | | 1.000 | | 0.56 | | 0.56 |
| SES FRic | ~ log(MAT) | 4 | | 93.52 | | 0.000 | | 0.706 | | 0.10 | | 0.52 |
|  | ~ none | 3 | | 95.28 | | 1.752 | | 0.294 | | 0.00 | | 0.35 |
| SES Hyp | ~ log(MAT) | 4 | | 90.66 | | 0.000 | | 1.000 | | 0.17 | | 0.17 |
| SES FEve | ~ log(TS) | 4 | | 81.80 | | 0.000 | | 0.432 | | 0.29 | | 0.42 |
|  | ~ log(MAT)+log(TS) | 5 | | 82.57 | | 0.772 | | 0.294 | | 0.30 | | 0.50 |
|  | ~ log(MAT) | 4 | | 82.72 | | 0.915 | | 0.274 | | 0.11 | | 0.47 |
| SES mNND | ~ none | 3 | | 80.02 | | 0.000 | | 0.664 | | 0.00 | | 0.00 |
|  | ~ log(PS) | 4 | | 81.38 | | 1.360 | | 0.336 | | 0.05 | | 0.05 |
| SES sdNND | ~ none | 3 | | 77.40 | | 0.000 | | 0.603 | | 0.00 | | 0.00 |
|  | ~ log(TS) | 4 | | 78.24 | | 0.838 | | 0.397 | | 0.07 | | 0.07 |

Table S6. Variable importance of environmental factors and species richness for five functional diversity indices and their standardized effect size (SES). Values were obtained from the best linear mixed models (LMM) explaining fern and lycophyte functional diversity across four elevational transects, weighted on AICc values (ΔAICc<2). LMM used a set of different explanatory variables based on species richness, one microclimatic variable (bryophyte cover) and four macroclimatic variables (mean annual temperature MAT, temperature seasonality TS, annual precipitation AP, and precipitation seasonality PS), using the transects as a random factor. The functional diversity indices characterize the volume as functional volume (FRic) and hypervolume (Hyp), evenness (FEve), and density as the mean (mNND) and the standard deviation (sdNND) of the nearest neighbor distance.

| General | log(sp_rich) | Bryophyte cover | log(MAT) | log(TS) | log(AP) | log(PS) |
| --- | --- | --- | --- | --- | --- | --- |
| log(FRic) | 0.61 | 0 | 0.1 | 0 | 0.2 | 0.1 |
| Hyp | 0.62 | 0.28 | 0 | 0 | 0 | 0.1 |
| FEve | 0.49 | 0.34 | 0.1 | 0 | 0.07 | 0 |
| log(mNND) | 0.78 | 0 | 0 | 0 | 0.22 | 0 |
| log(sdNND) | 0.28 | 0.28 | 0.28 | 0.09 | 0 | 0.09 |
| SES FRic | - | 0 | 0 | 0 | 0 | 1.00 |
| log(SES Hyp) | - | 1.00 | 0 | 0 | 0 | 0 |
| SES FEve | - | 0.26 | 0 | 0.38 | 0.06 | 0.30 |
| SES mNND | - | 0 | 0 | 0 | 0 | 1.00 |
| SES sdNND | - | 0.33 | 0.33 | 0.33 | 0 | 0 |

Table S7. Variable importance of environmental factors and species richness in epiphytes for five functional diversity indices and their standardized effect size (SES). Values were obtained from the best linear mixed models (LMM) explaining epiphytic fern and lycophyte functional diversity across four elevational transects, weighted on AICc values (ΔAICc<2). LMM used a set of different explanatory variables based on species richness, one microclimatic variable (bryophyte cover) and four macroclimatic variables (mean annual temperature MAT, temperature seasonality TS, annual precipitation AP, and precipitation seasonality PS), using the transects as a random factor. The functional diversity indices characterize the volume as functional volume (FRic) and hypervolume (Hyp), evenness (FEve), and density as the mean (mNND) and the standard deviation (sdNND) of the nearest neighbor distance.

| Epiphytes | log(sp_rich) | Bryophyte cover | log(MAT) | log(TS) | log(AP) | log(PS) |
| --- | --- | --- | --- | --- | --- | --- |
| log(FRic) | 0.63 | 0 | 0 | 0.25 | 0 | 0.13 |
| Hyp | 0.51 | 0 | 0 | 0.14 | 0 | 0.36 |
| FEve | 0.34 | 0 | 0.16 | 0.34 | 0.11 | 0.05 |
| log(mNND) | 0.48 | 0.07 | 0.11 | 0 | 0.35 | 0 |
| sdNND | 0.44 | 0.44 | 0 | 0 | 0.13 | 0 |
| SES FRic | - | 0.27 | 0.23 | 0.25 | 0 | 0.25 |
| SES Hyp | - | 0.37 | 0.44 | 0 | 0 | 0.19 |
| SES FEve | - | 0.18 | 0.41 | 0.41 | 0 | 0 |
| SES mNND | - | 0 | 0 | 0 | 0 | 1.00 |
| SES sdNND | - | 0.20 | 0.24 | 0 | 0.56 | 0 |

Table S8. Variable importance of environmental factors and species richness in terrestrials for five functional diversity indices and their standardized effect size (SES). Values were obtained from the best linear mixed models (LMM) explaining terrestrial fern and lycophyte functional diversity across four elevational transects, weighted on AICc values (ΔAICc<2). LMM used a set of different explanatory variables based on species richness, one microclimatic variable (bryophyte cover) and four macroclimatic variables (mean annual temperature MAT, temperature seasonality TS, annual precipitation AP, and precipitation seasonality PS), using the transects as a random factor. The functional diversity indices characterize the volume as functional volume (FRic) and hypervolume (Hyp), evenness (FEve), and density as the mean (mNND) and the standard deviation (sdNND) of the nearest neighbor distance.

| Terrestrials | log(sp_rich) | Bryophyte cover | log(MAT) | log(TS) | log(AP) | log(PS) |
| --- | --- | --- | --- | --- | --- | --- |
| log(FRic) | 0.66 | 0 | 0.18 | 0 | 0 | 0.16 |
| Hyp | 0.4 | 0 | 0.4 | 0 | 0 | 0.2 |
| FEve | 0.39 | 0 | 0.21 | 0.39 | 0 | 0 |
| log(mNND) | 1 | 0 | 0 | 0 | 0 | 0 |
| sdNND | 1 | 0 | 0 | 0 | 0 | 0 |
| SES FRic | - | 0 | 1 | 0 | 0 | 0 |
| SES Hyp | - | 0 | 1 | 0 | 0 | 0 |
| SES FEve | - | 0 | 0.44 | 0.56 | 0 | 0 |
| SES mNND | - | 0 | 0 | 0 | 0 | 1 |
| SES sdNND | - | 0 | 0 | 1 | 0 | 0 |


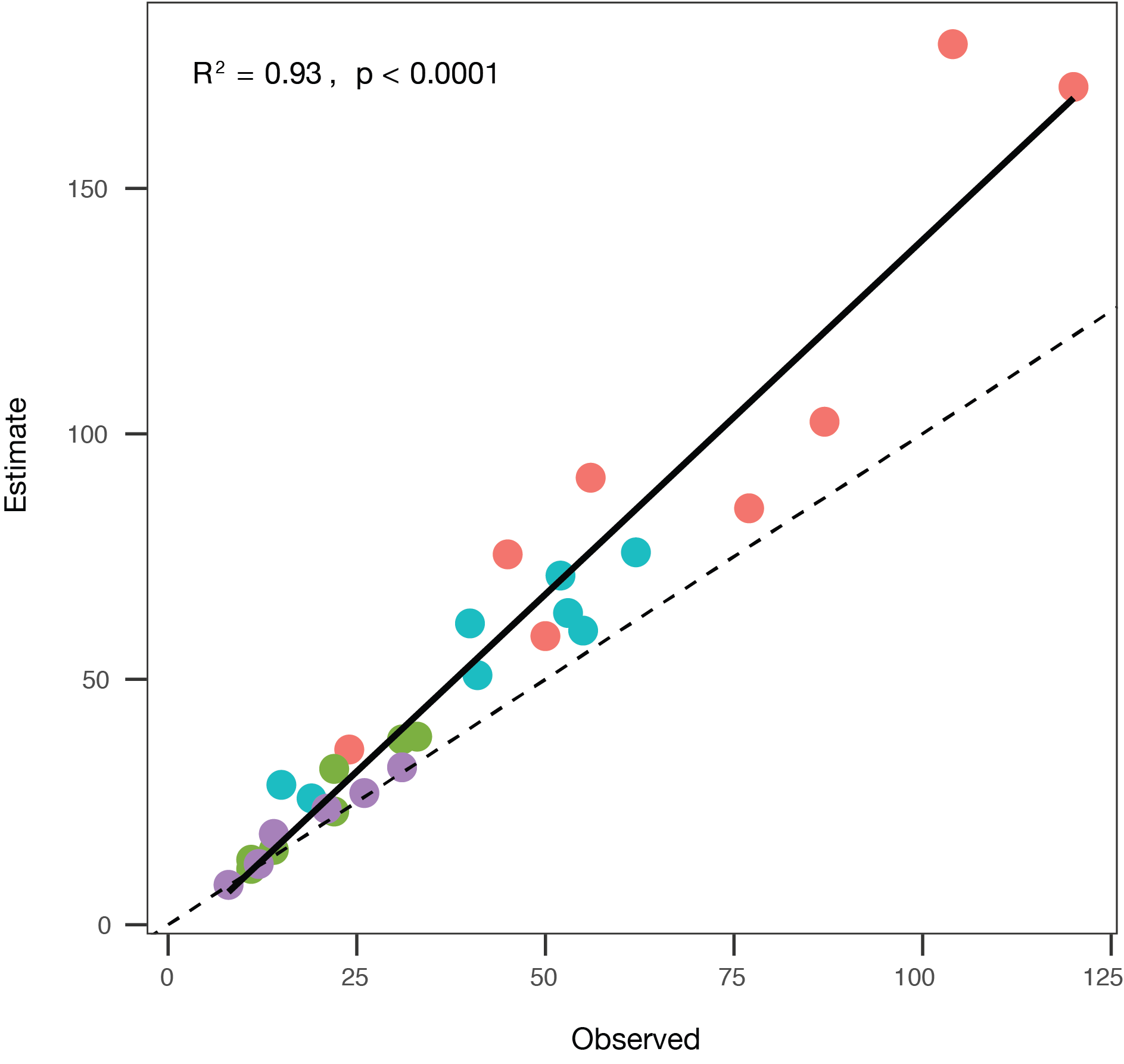


Fig. S1. Linear regressions of observed versus estimated species richness of ferns and lycophytes along four elevational transects in the tropics, computed with Chao2 estimator. The dotted line represents the middle, and the black line the general trend. The transects are Ecuador (red), Mexico (green), Papua New Guinea (blue), and Uganda (purple).


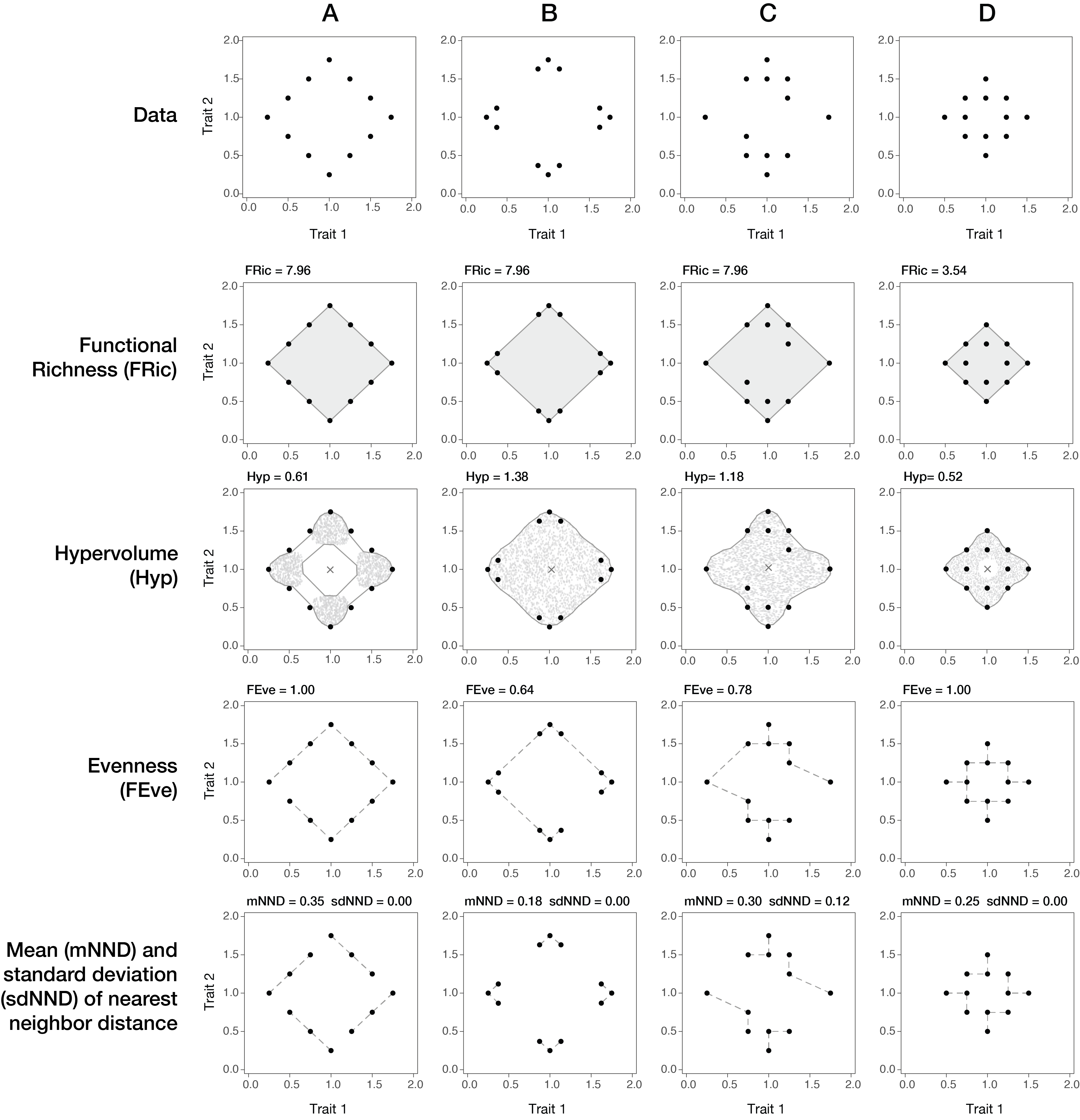


Fig. S2. Graphic representation of the five functional diversity indices in a functional space used in this study. The upper panels show four different fictional communities (a-d) with 12 species placed in trait space in relation to 2 traits. The four rows below show the aspects of functional diversity captured by each index and how the indices vary in response to the distribution of the species in trait space.


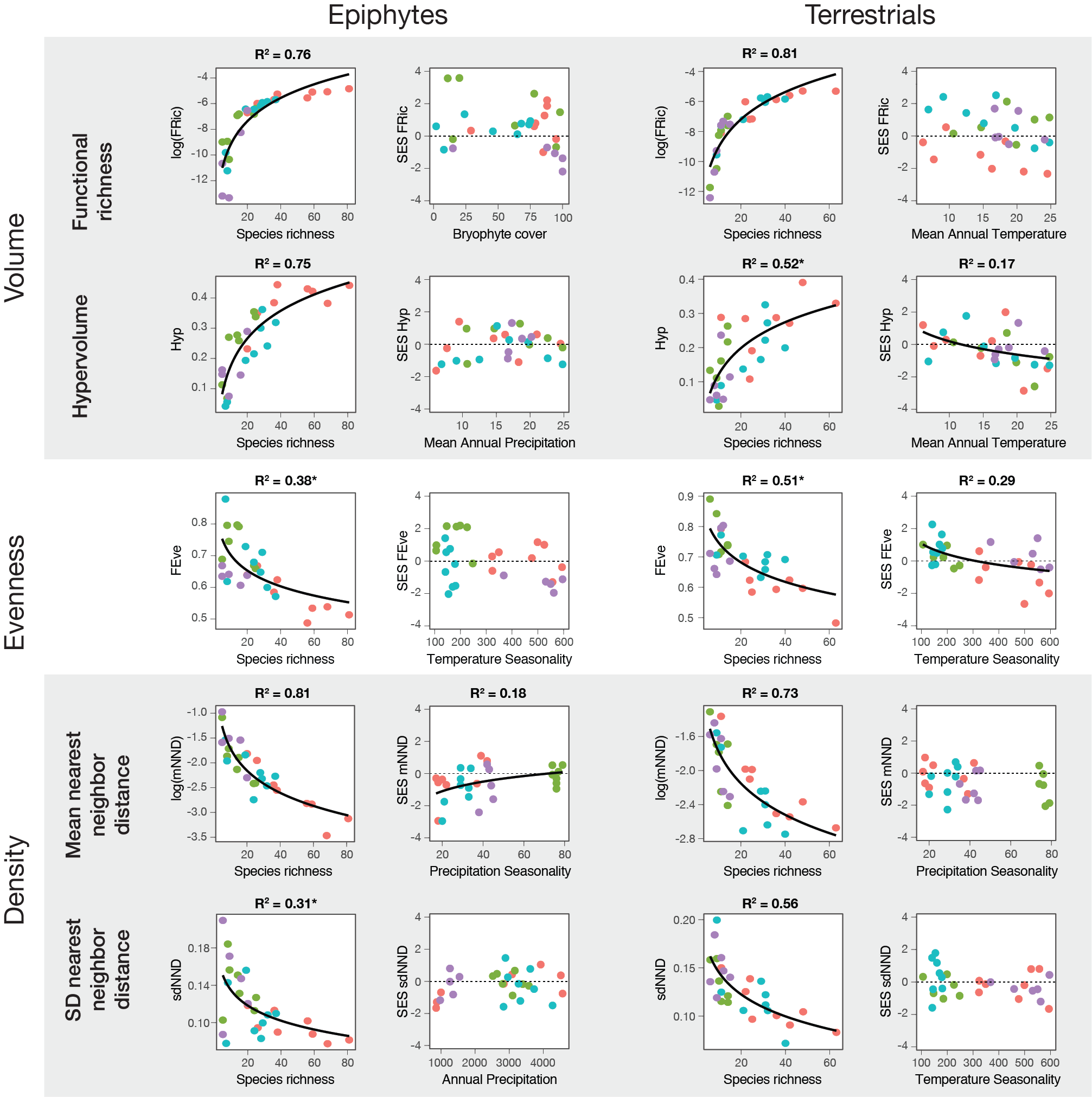


Fig. S3. Functional diversity indices of ferns and lycophytes in relation to species richness and environmental variables for four tropical transects in Ecuador (red), Mexico (green), Papua New Guinea (blue), and Uganda (purple). Linear mixed models (LMM) were used to contrast climate data and species richness against all indices, as well as the standardized effect sizes (SES) of each index using the transects as a random factor. The LMMs were calculated separately for epiphytic and terrestrial species. Climatic data used was one microclimatic variable (bryophyte cover) and four macroclimatic variables (mean annual temperature, temperature seasonality, annual precipitation, and precipitation seasonality). All R^2^ values are of LMM containing only species richness or the climatic data specified in the graph and were part of the selected models for the variable importance analysis (best fitting models, ΔAICc<2). In contrast, values marked with (*) are LMM that were not selected but still have significantly explained variance.


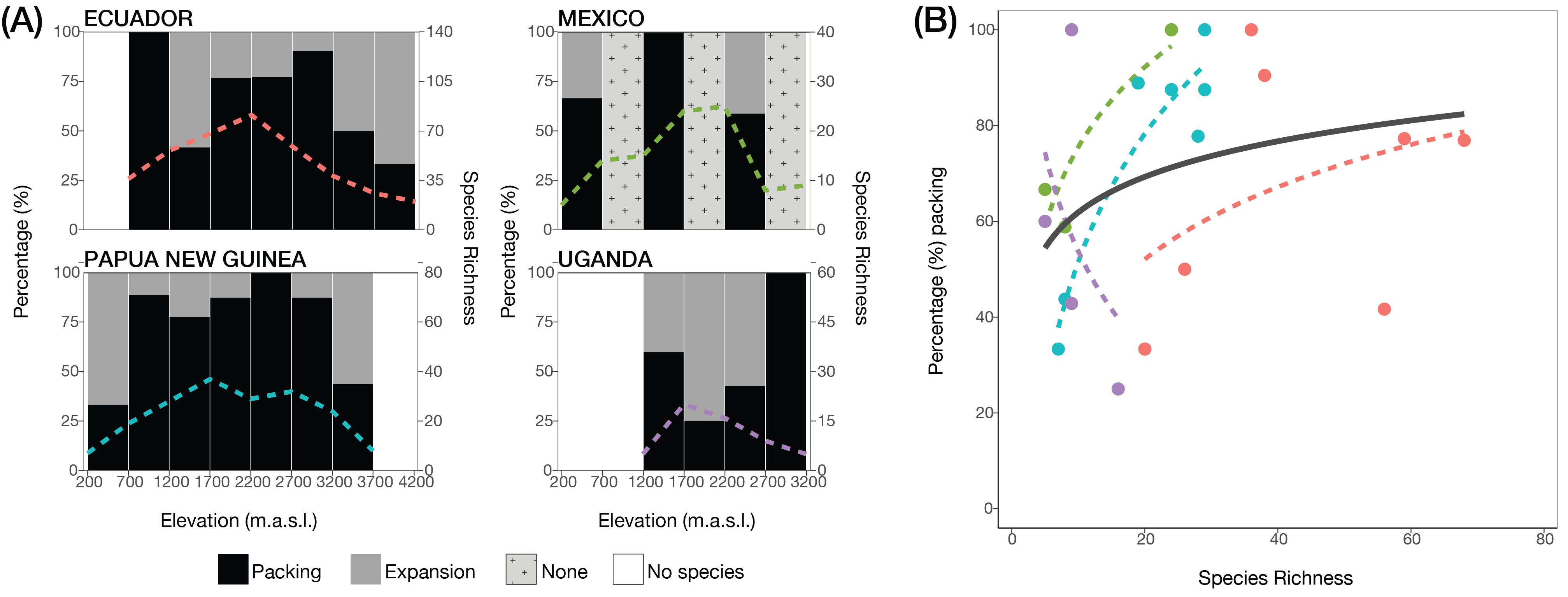


Fig. S4. Niche packing and expansion of epiphytic ferns along four elevational gradients. (a) The percentage contribution of packing (black) and expansion (grey) between adjacent elevational bands, showing species richness by the colored dotted lines. If there is no difference in species richness from one elevation to the other, then no index was not calculated and the step appears in light grey as none. (b) The relation of packing percentage with the increase of species richness for all transects (ΔAICc=3.49 χ^2^(1)=11.4, p<0.001). The transects are indicated by colors for Ecuador (red), Mexico (green), Papua New Guinea (blue), and Uganda (purple). The general trend is given in black and the transects are shown by the same color as in (a).


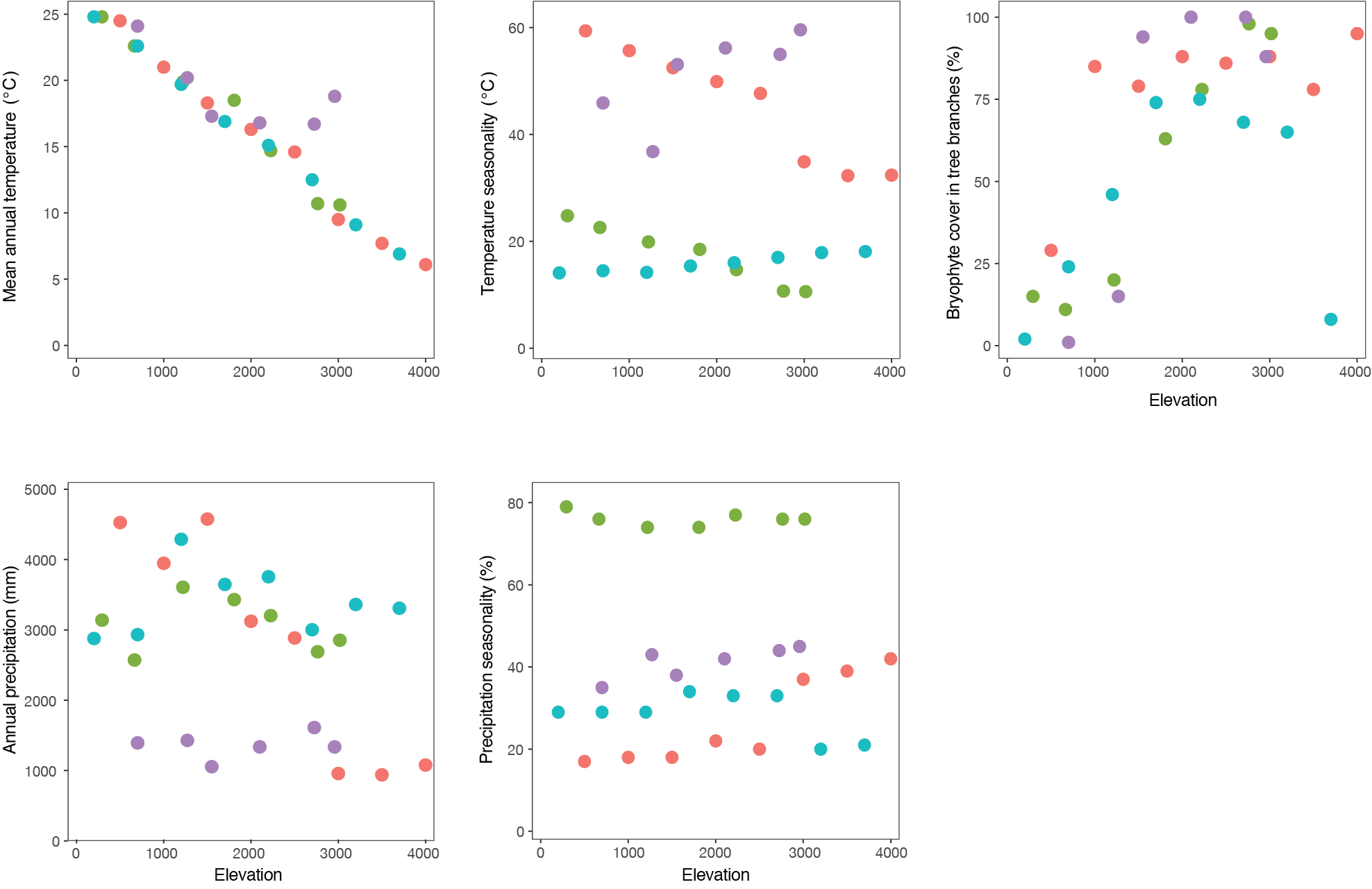


Fig. S5. Climatic variables of the four elevational transects studied in Ecuador (red), Mexico (green), Papua New Guinea (blue), and Uganda (purple). There are four macroclimatic variables (a-d) and one microclimatic variable (e).
